# Supplementary material for: Doxorubicin induces caspase-mediated proteolysis of KV7.1
Source: Commun Biol. 2018 Sep 28;1:155. doi: 10.1038/s42003-018-0162-z (PMC6162258; doi:10.1038/s42003-018-0162-z)

Supplementary Information for

Doxorubicin induces caspase-mediated proteolysis of $K_v7.1$

Anne Strigli, Christian Raab, Sabine Hessler, Tobias Huth, Adam J. T. Schuldt,
Christian Alzheimer, Thomas Friedrich, Paul W. Burridge,
Mark Luedde and Michael Schwake

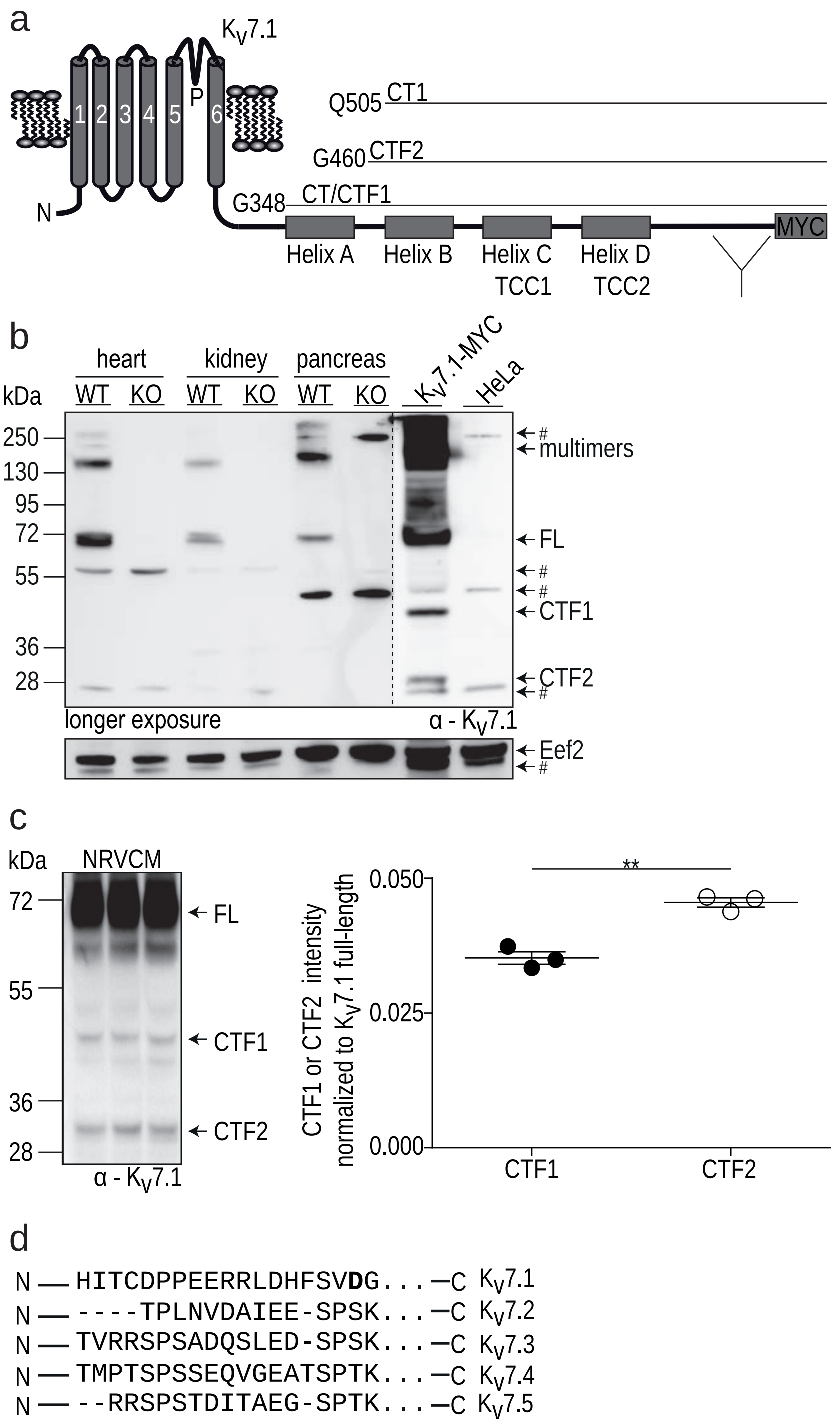

Supplementary Figure 1: a, Schematic illustration of the topology of Kv7.1. Voltage Sensing Domain (S1 – S4) and Pore Domain (S5 – S6) are shown within the membrane. The C-terminus contains four helices. The third (helix C) and fourth (helix D) helices are also known as tetramerization coiled-coil 1 and 2 for their high probability to form amphipathic helical structures. Wye indicates binding site of the C-terminal anti-Kv7.1 antibody to the last eleven amino acids. Assumed starting points of CT- and CT1-construct as well as CTF1 and CTF2 are highlighted. **b,** Western blot analysis of indicated mouse organs. Untransfected (Ø) and HeLa cells overexpressing Kv7.1 as well as Kv7.1 deficient tissue lysates served as negative controls. # indicates nonspecific binding of the antibody. **c,** Lysates of NRVM were analyzed by immunoblotting. Densitometric analysis of 3 independent experiments of the CTF1 or CTF2 band intensity normalized to the Kv7.1 full-length band intensity. Statistics were tested with two-tailed unpaired student's t-test. **d,** Alignment of Kv7.1 – Kv7.5. The D459 in Kv7.1 is highlighted in bold. **(b,c)** anti-Kv7.1 antibody, anti-Eef2 antibody. All dot blots are shown as mean and error bars as SEM.

Species	Amino acid sequence
Homo sapiens	H I T C D P - P - - E E R R L D H F S V D G
Chelonia mydas	Q I T Y D H V M - - D D R K S D - F N I D A
Rattus norvegicus	H I T C D P - P - - E D R R P D H F S I D G
Heterocephalus glaber	H I T C D P - P - - E E R R P D H F S V D G
Canis familiaris	H I T C E P V S - - E E R R P D H F S V D N
Felis catus	H I T C E P V S - - E K R R P D H F S V D T
Squalus acanthias	N I T Y D H V V D K D D R K F E N S N I D G
Loxodonta africana	H I T Y D P V G - - D D R R A D R L S L D G
Anolis carolinensis	Q I T F D H A V - - D E R K S D N F H L D P
Chrysemys picta bellii	H I T Y D H V M - - D D R K S D - F N I D A
Pelodiscus sinensis	H I T Y D H V V - - D D R K S D - F S I D A
Ovis aries	H I T C E P V L - - E D R R P E P I S V D G
Mus musculus	H I T Y D P - P - - E D R R P D H F S I D G
Sorex araneus	H I T C E P P A - - E G R R P V H F S V D G
Dasypus novemcinctus	H I T C E P A P - - E E R R P D H A S L D G
Meleagris gallopavo	H I T Y D H V T - - D D K K P D - F C F D T
Pantholops hodgsonii	H I T C E P V L - - X D R R P E P V S V D G
Vicugna pacos	H I T C D L A S - - E E R R P D H F S V D G
Bos taurus	H I T C D P V A - - E D R R P E P F S V D G
Pongo abelii	H I T C D P - P - - E E R R L D H F S V D G
Tursiops truncatus	H I T C D P T S - - E E R R P E H F S V D G
Trichechus manatus	H I T C D P A G - - E D R R A D G L S L D G
Papio anubis	H I T C D P - P - - E E R R L D H F S V D G
Camelus ferus	H I T C D L A A - - E E R R P D H F S V D G
Gallus gallus	H I T Y D H V T - - D D K K P D - F C F D T
Sarcophilus harrisii	H I T Y D H I A - - E D R K S D Q F A M D G
Echinops telfairi	Q I T C D A A C - - E E R R P D H S A L D G
Ochotona princeps	H I T C E P - P - - E E R R P D H F S T D S
Alligator mississippiensis	H I T Y D H V M - - D D R K S D - F S I D A
Latimeria chalumnae	H I T Y D H V M - - N D P K S E G L S M D G
Cricetulus griseus	H I T Y D P - P - - E D R R P D H F S I D G
Macaca mulatta	H I T C D P - P - - E E R R L D H F S V D G
Mustela putorius furo	H I T C E P V S - - E D R K P D H F S V D S
Pteropus alecto	H I T C D H V P - - D E Q R T D P F S V D C
Chinchilla lanigera	H I T C D P - P - - E E R R P D H F S V D G

Supplementary Figure 2: Alignment of K_V7.1 from 35 different species. The D459 is highlighted in red.

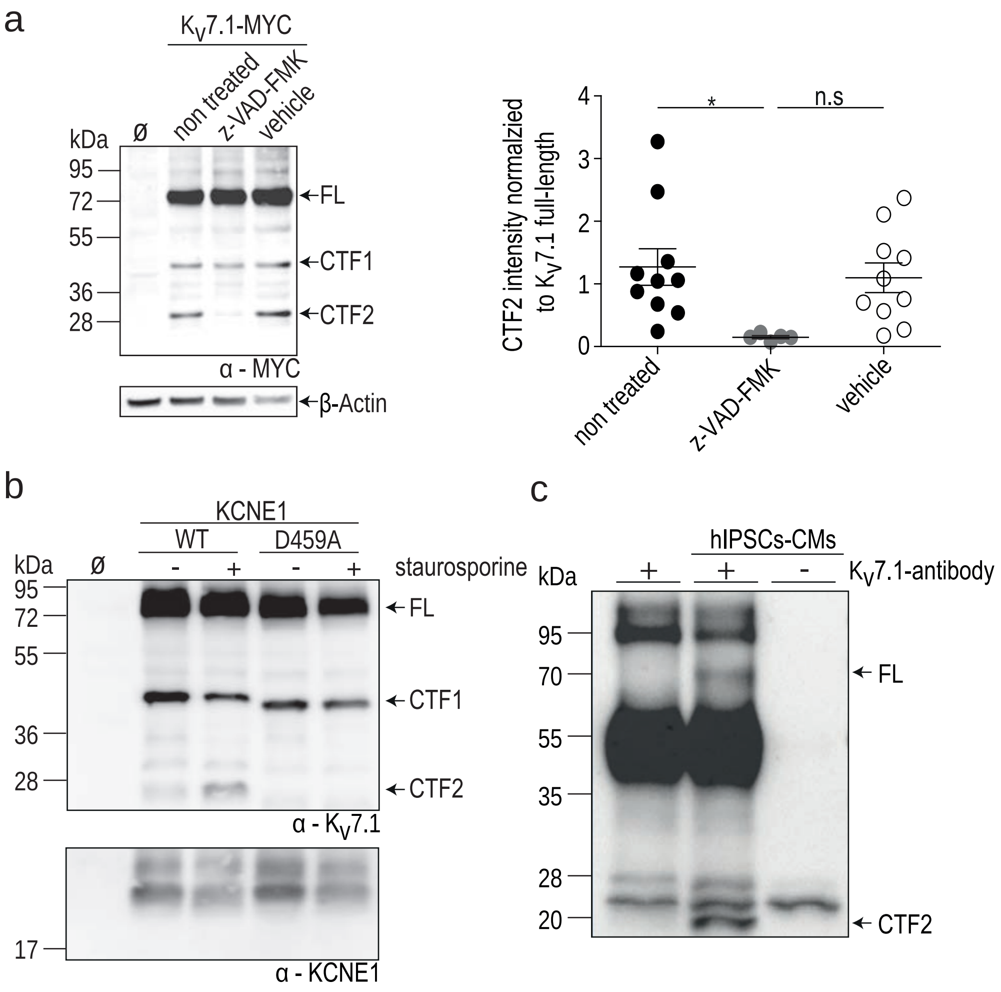

Supplementary Figure 3: a, Western blot analysis of Cos7 cells expressing Kv7.1-MYC treated with 50 – 100 µmol per L z-VAD-FMK for 12 hours. Untransfected (ø), non-treated and vehicle-treated cells served as negative controls. Densitometric analysis of 5 – 10 independent experiments of CTF2 band intensity normalized to Kv7.1 full-length band intensity. Statistics were tested with One-Way Anova followed by Bonferroni's Multiple Comparison Test. **b,** Western blot analysis of cells used for patch-clamp recordings. **c,** Immunoprecipitation study analyzed by western blots of human induced pluripotent stem cell dervied cardiomyocytes with a Kv7.1 antibody. Lysate without antibody and antibody control served as negative controls. **(a)** anti-myc antibody, anti-β-actin antibody. **(b)** anti-KCNE1 antibody. **(b,c)** anti-Kv7.1 antibody. All dot blots are shown as mean and error bars as SEM.

Supplementary Information - Uncropped Blots

Figure 1a - α - K_V7.1

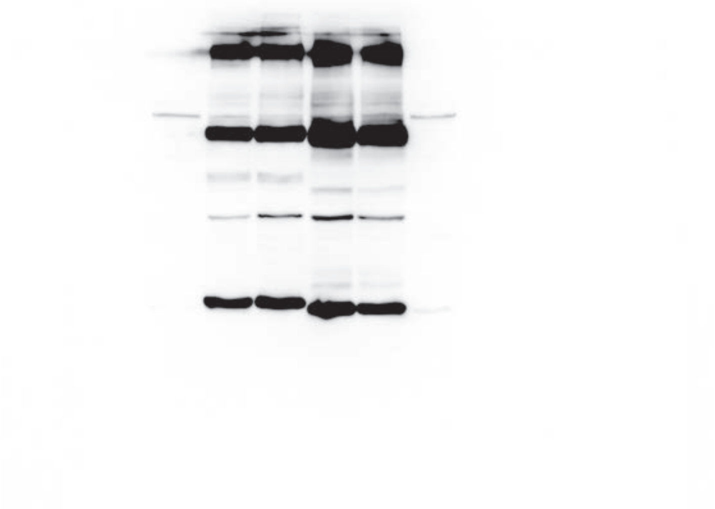

Figure 1b - α - MYC

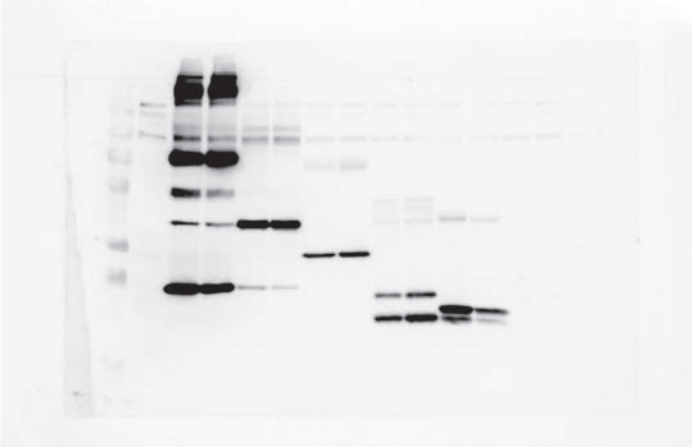

Figure 1c - α - MYC

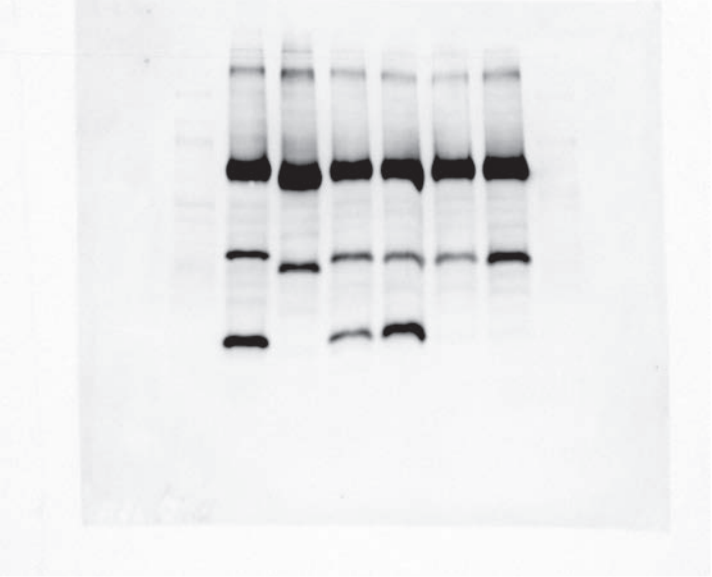

Figure 1d - α - MYC

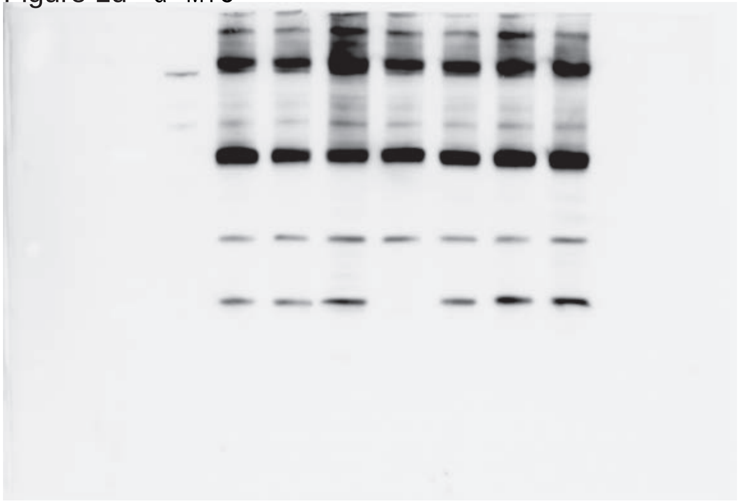

Figure 1d - α - actin

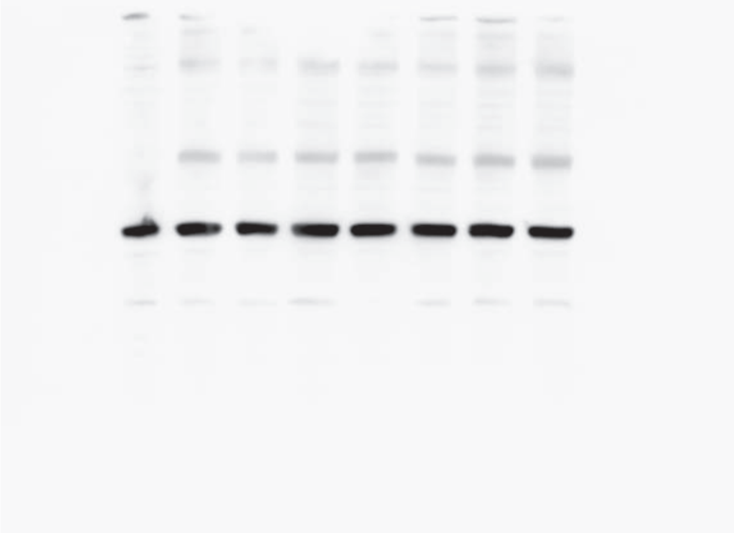

Figure 2a - α - MYC

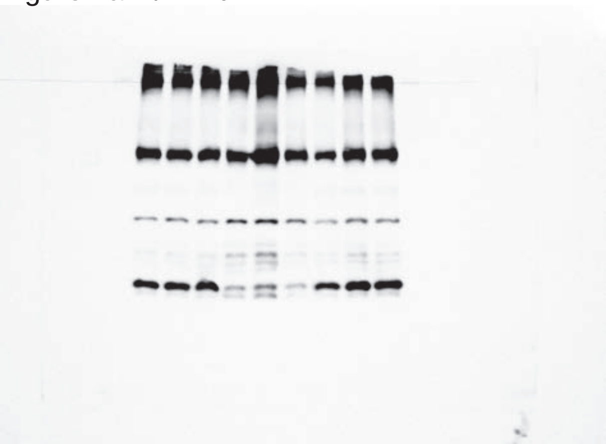

Figure 2b - α - MYC

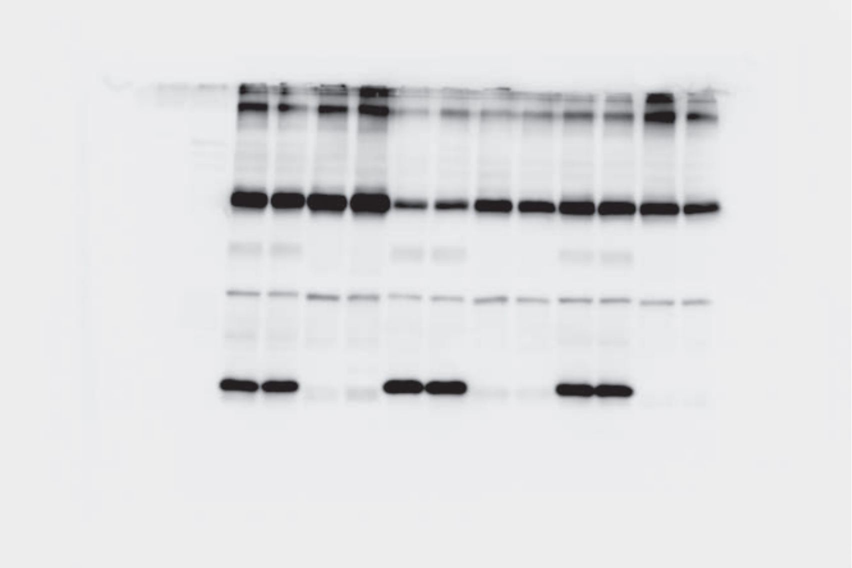

Figure 2c - α - MYC

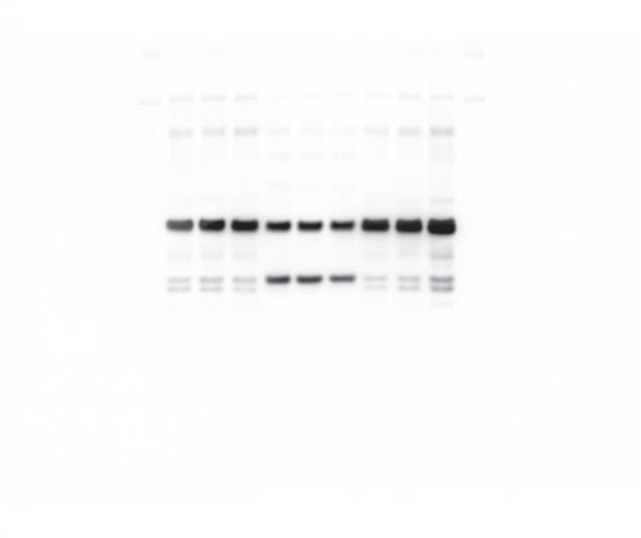

Figure 2c - α - actin

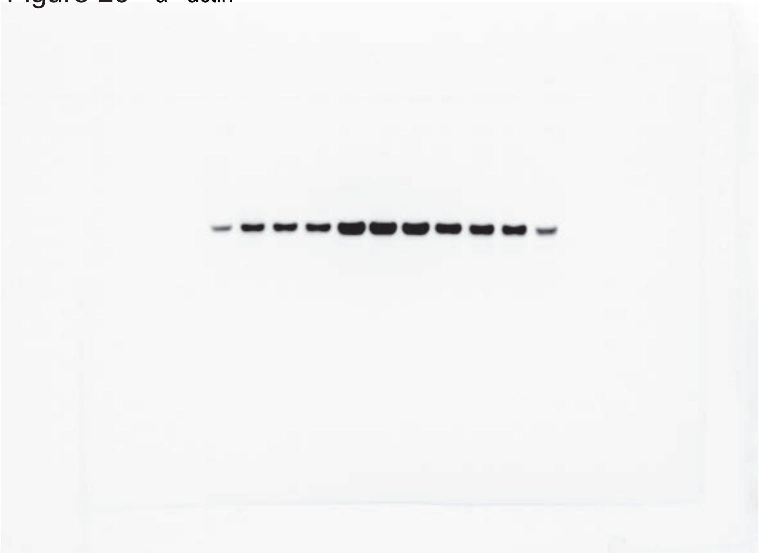

Figure 3a - α - MYC

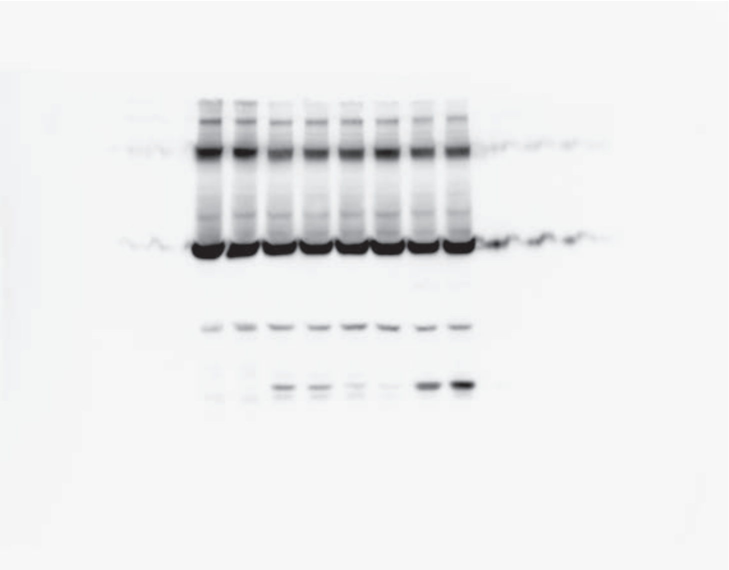

Figure 3a - α - caspase-3

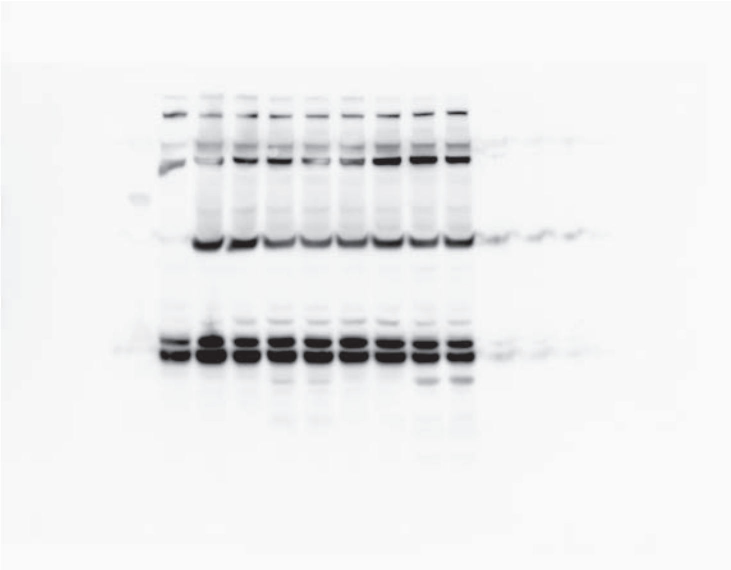

Figure 3a - α - actin

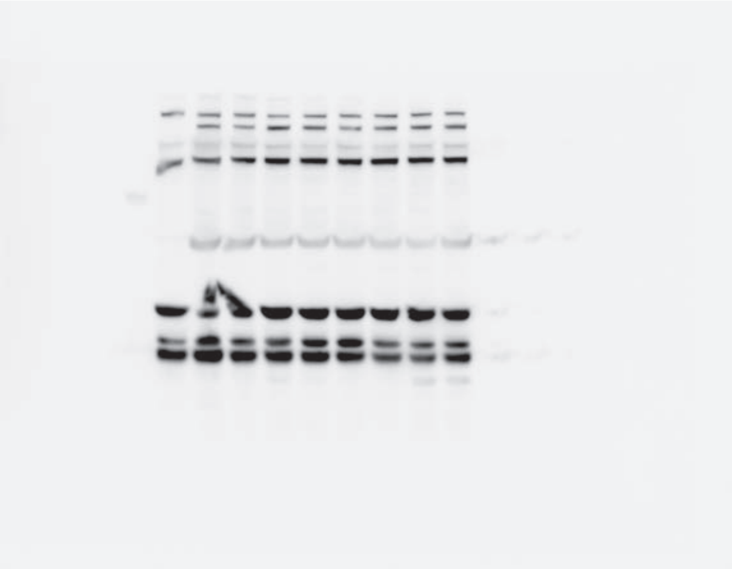

Figure 3b - α - MYC

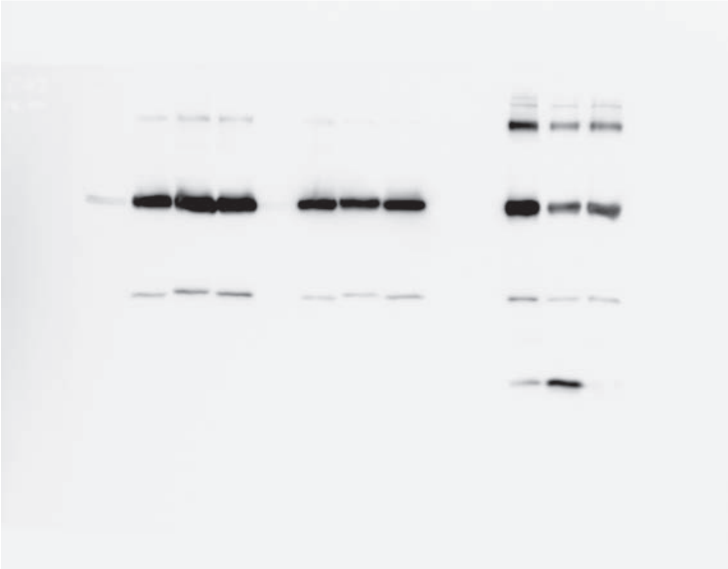

Figure 3b - α - caspase-3

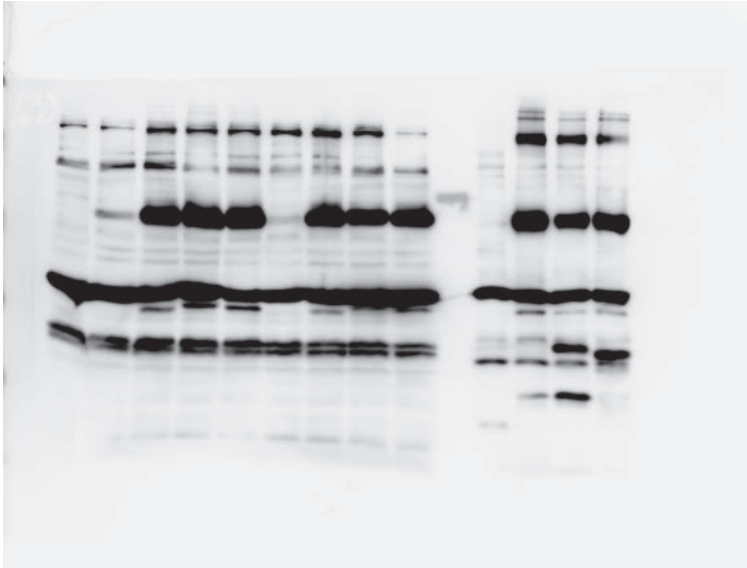

Figure 3b - α - actin

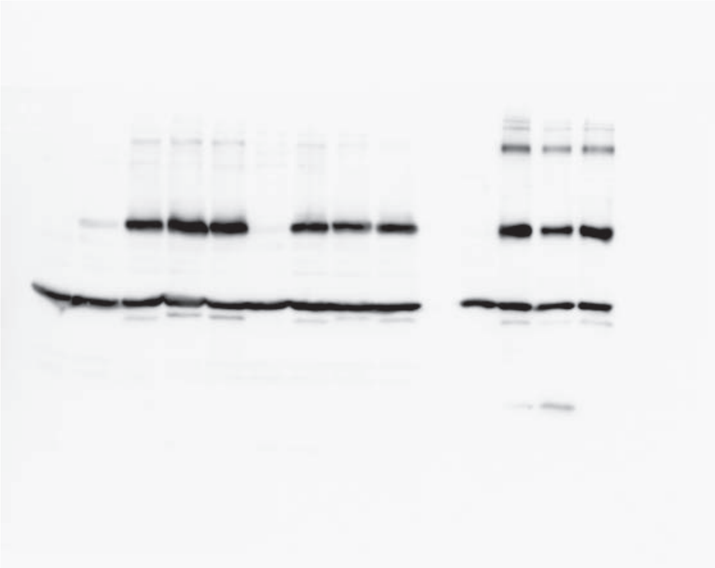

Figure 3c - α - MYC

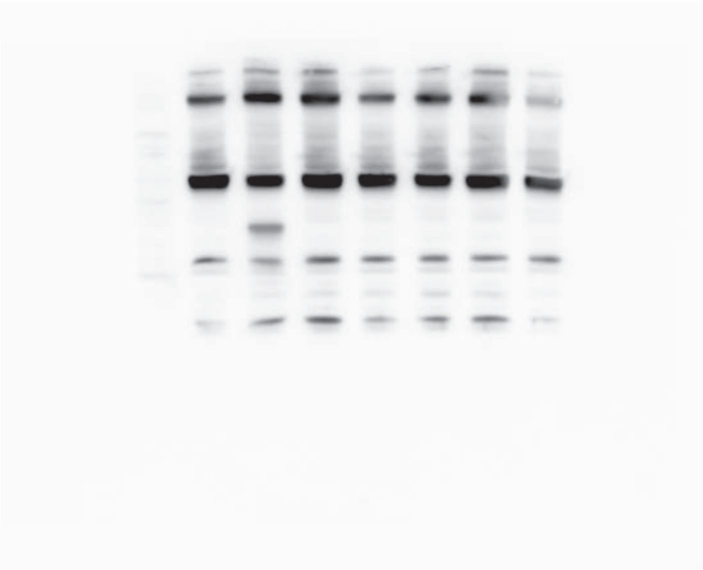

Figure 3c - α - actin

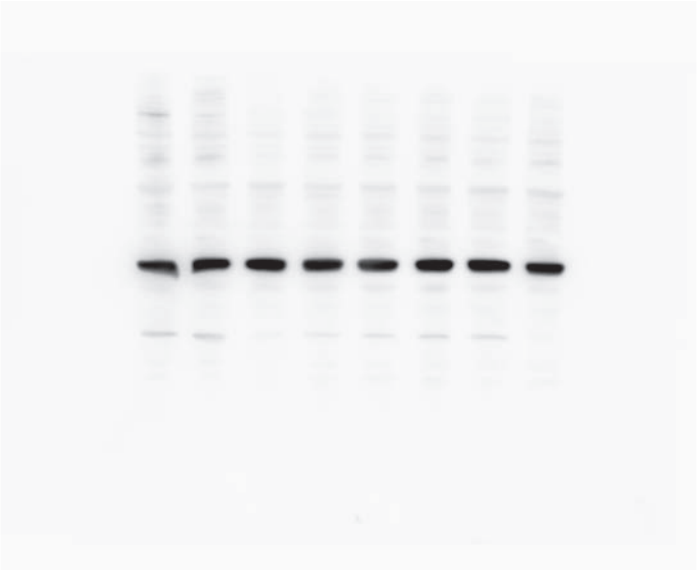

Figure 3d - α - K_V7.1

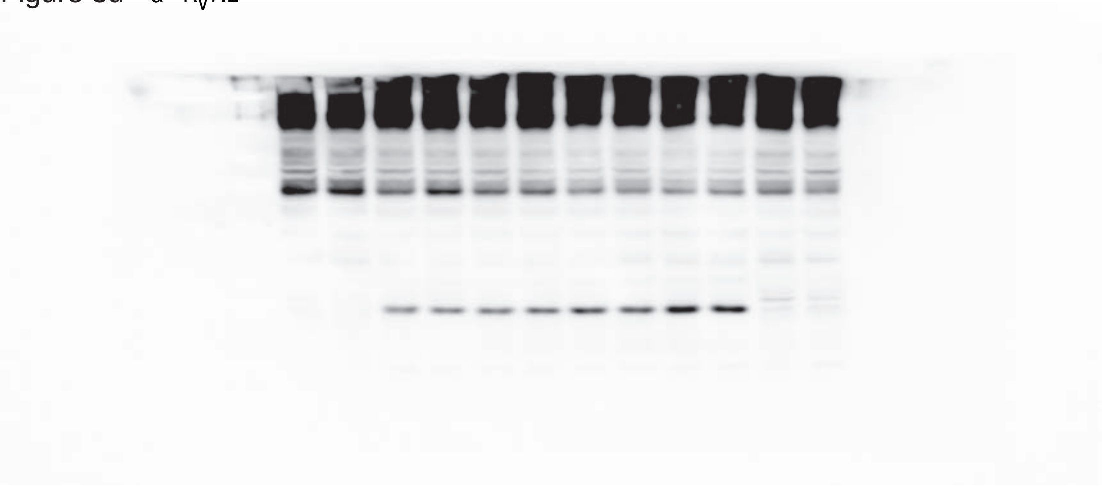

Figure 3d - α - caspase-3

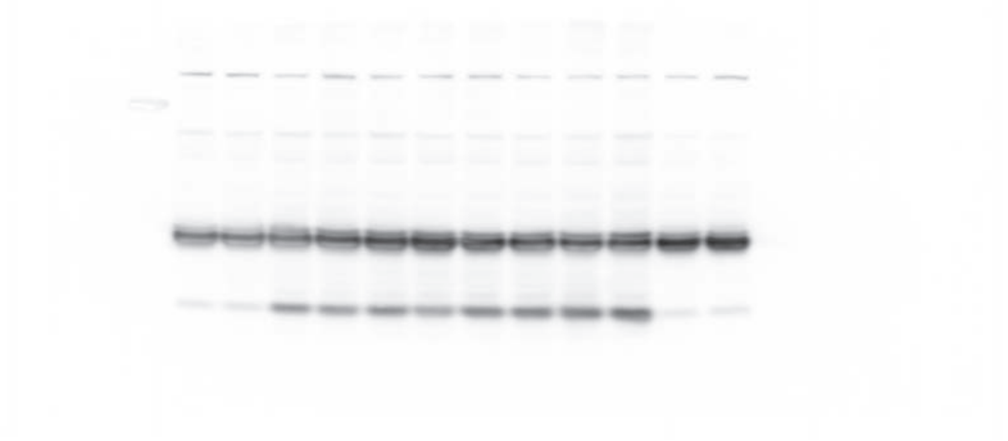

Figure 4c - α - K_V7.1

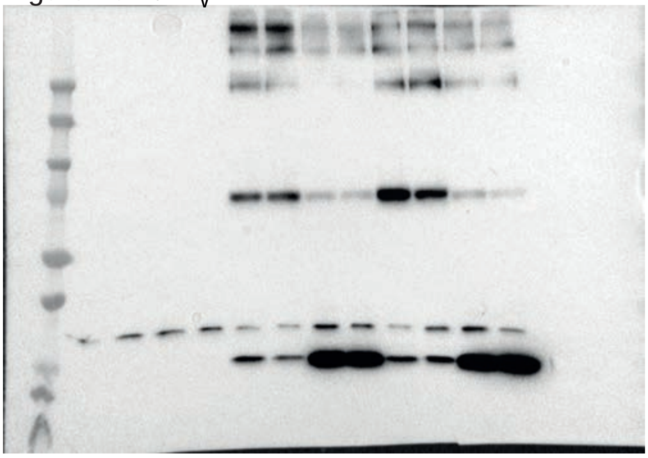

Figure 4c - α - KCNE1

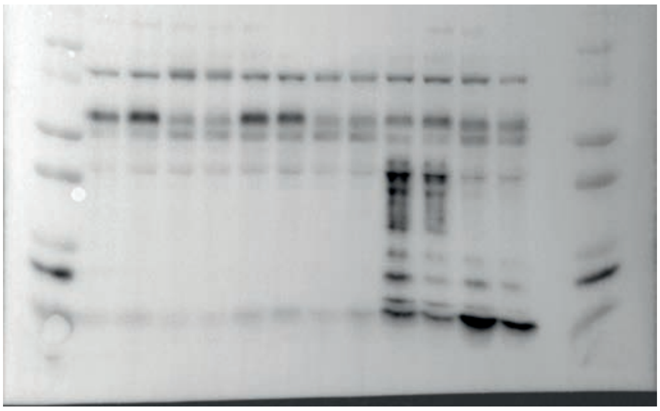

Figure 4c - α - GAPDH

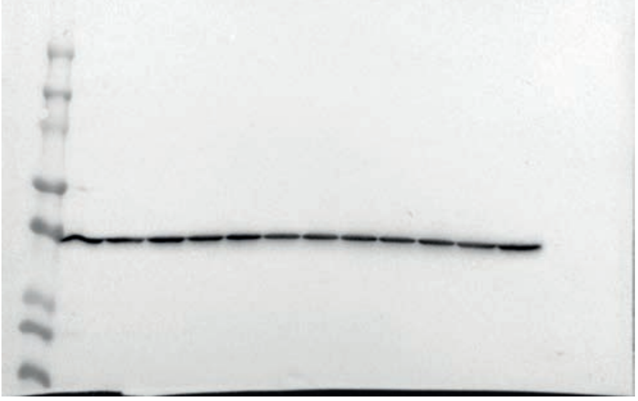

Figure 4c - α - caspase-3

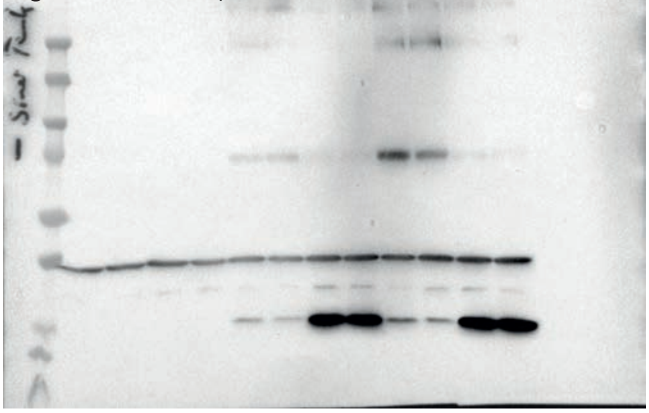

Figure 4d - α - K_V7.1 IP

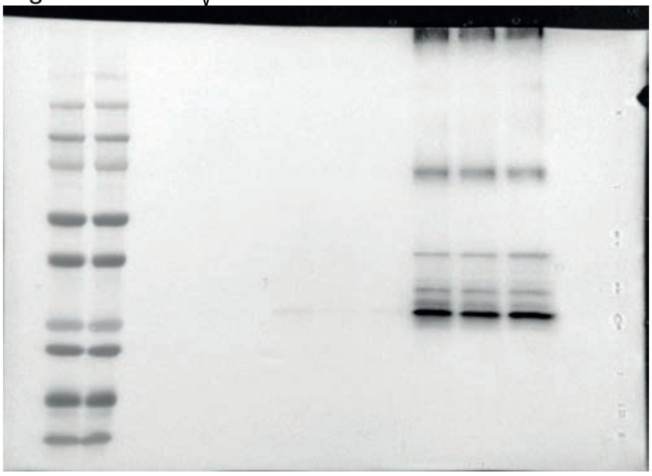

Figure 4d - α - K_V7.1 TL

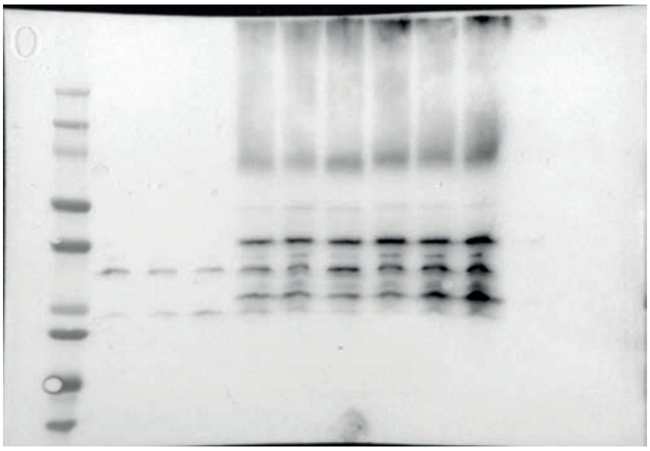

Figure 4d - α - GAPDH IP

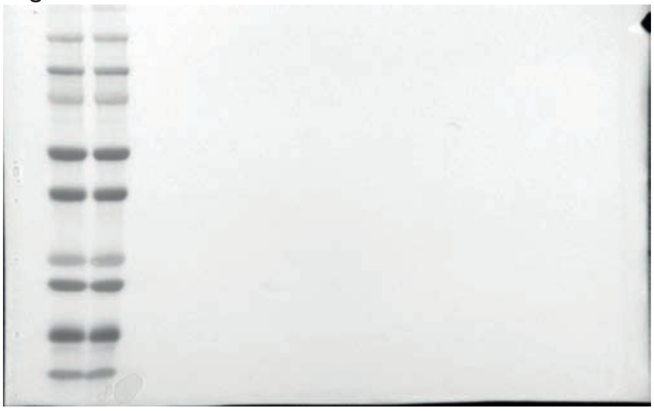

Figure 4d - α - GAPDH TL

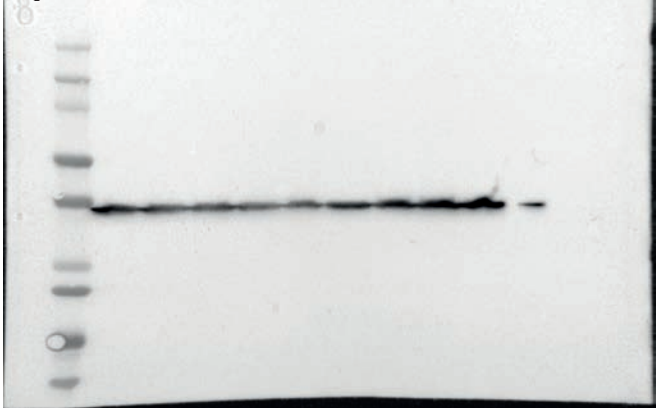

Figure 4e - α - MYC

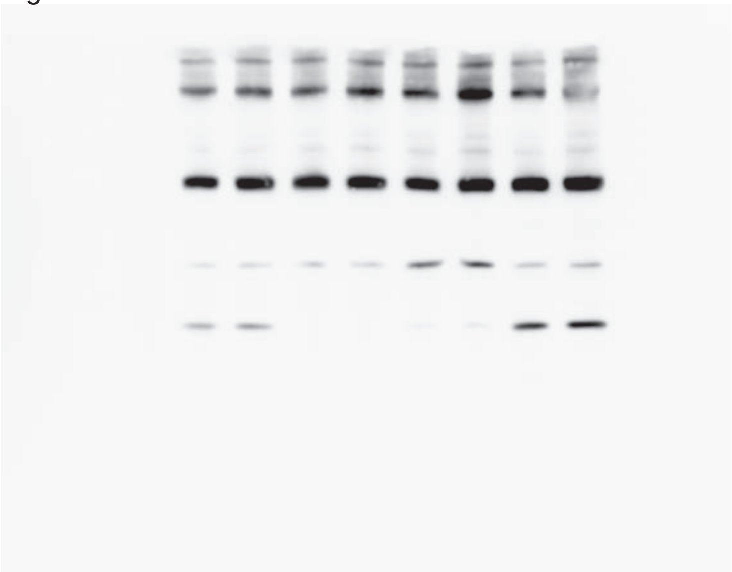

Figure 4e - α - actin

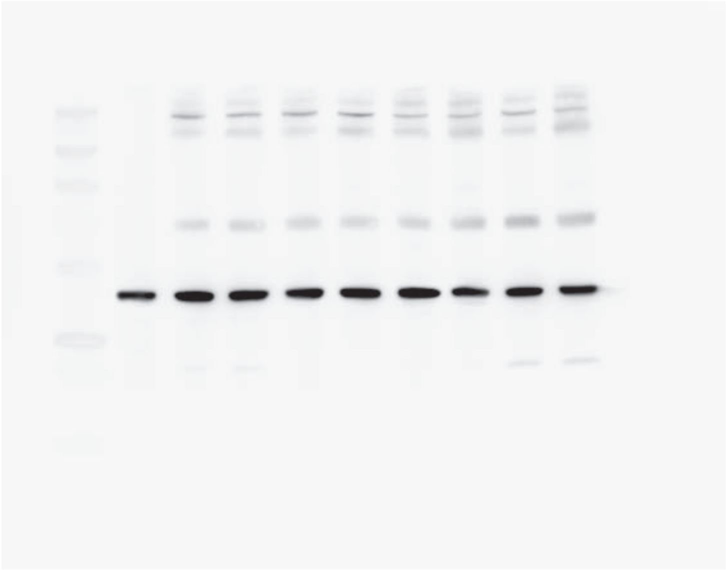

Figure 4f - α - MYC (right IP; left TL)

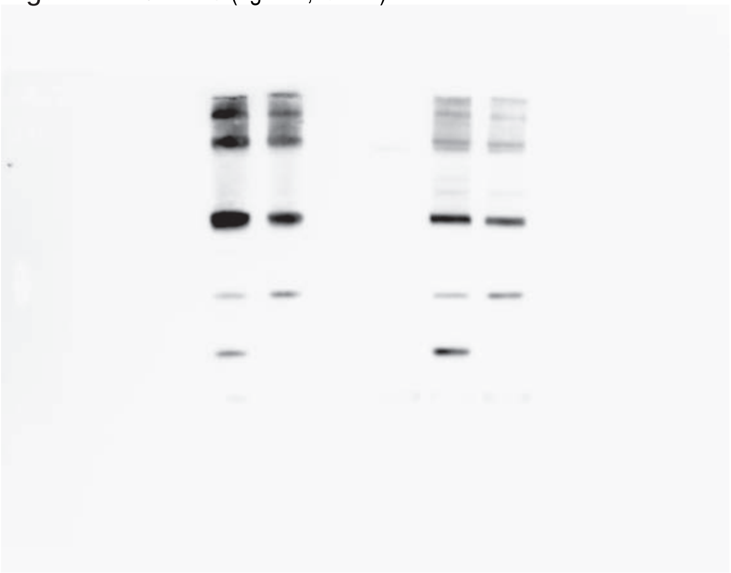

Figure 4f - α - calmodulin (right IP; left TL)

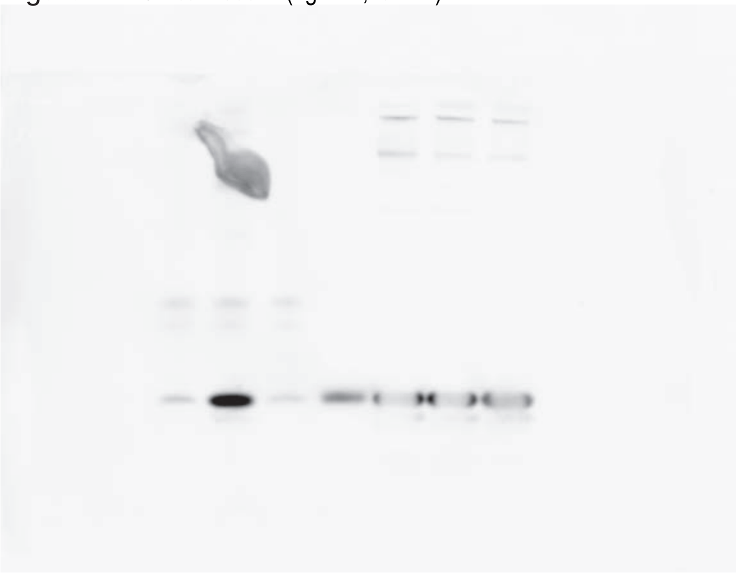

Figure 5a - α - $K_V7.1$

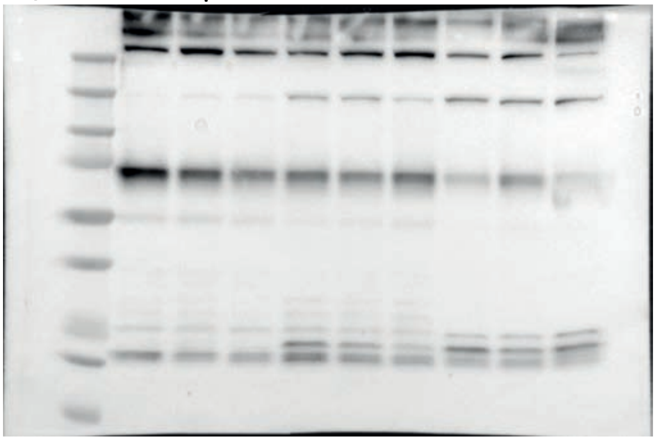

Figure 5a - α - GAPDH

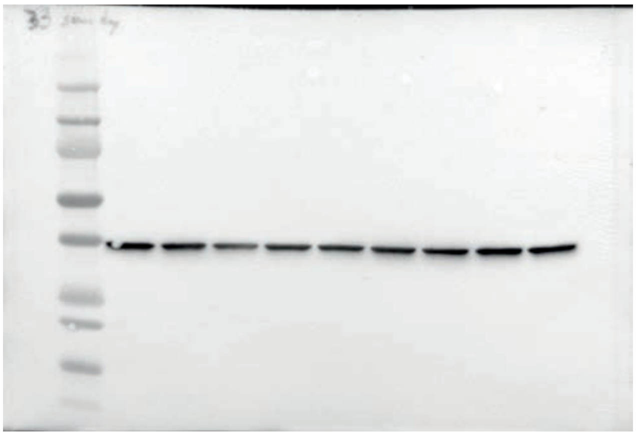

Figure 5a - α - caspase-3

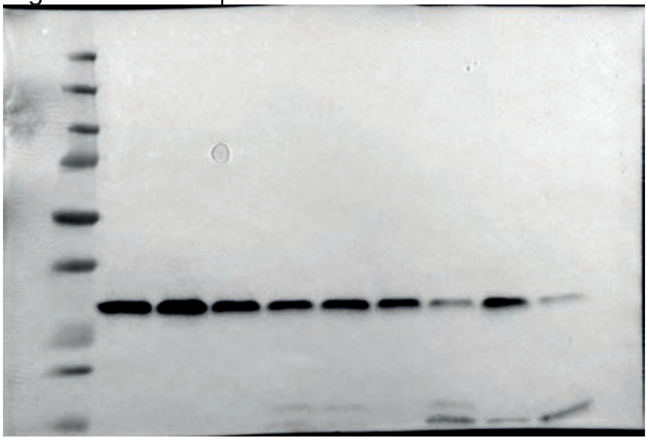

Supplementary Figure 1b - α - K_V7.1

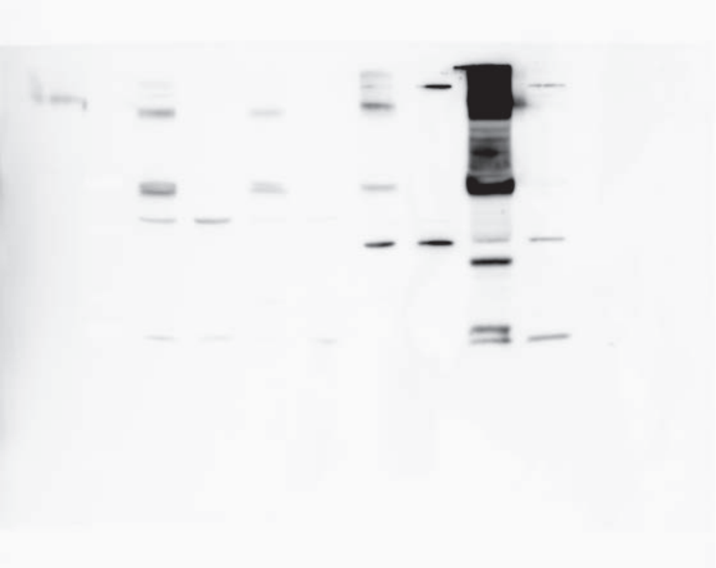

Supplementary Figure 1b - α - Eef2

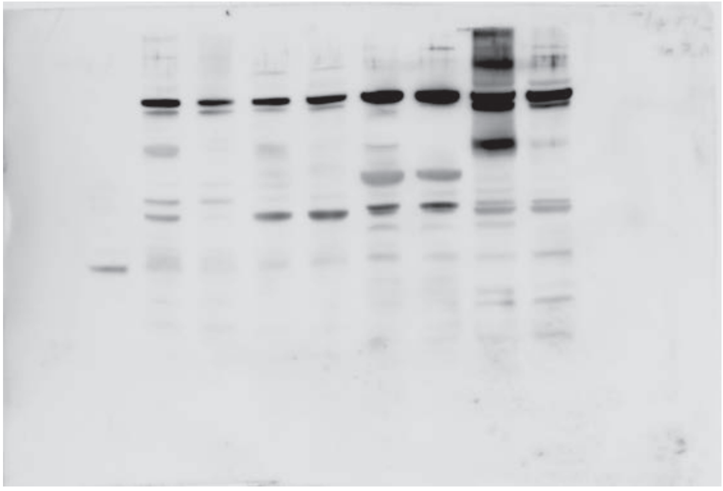

Supplementary Figure 1c - α - K_V7.1

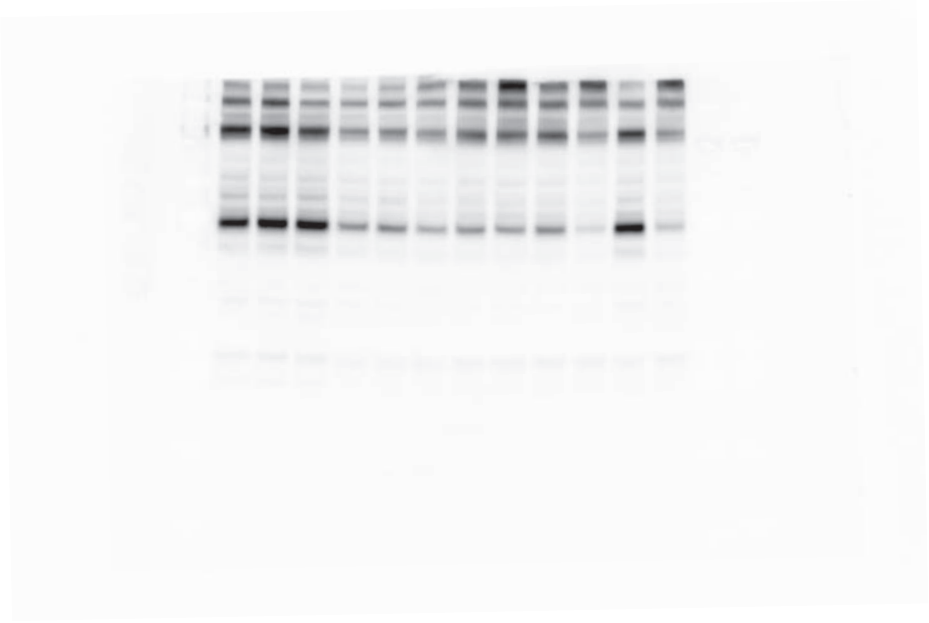

Supplementary Figure 3a - α - MYC

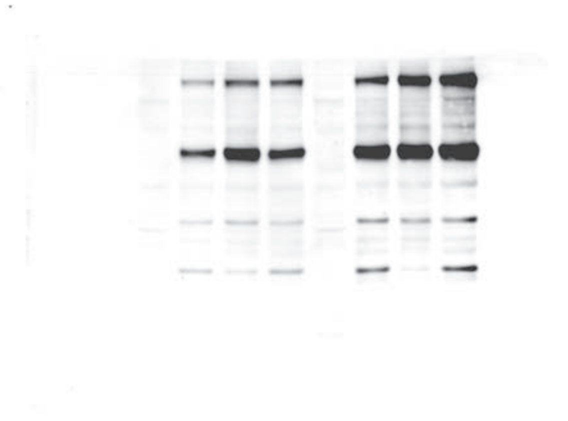

Supplementary Figure 3a - α - actin

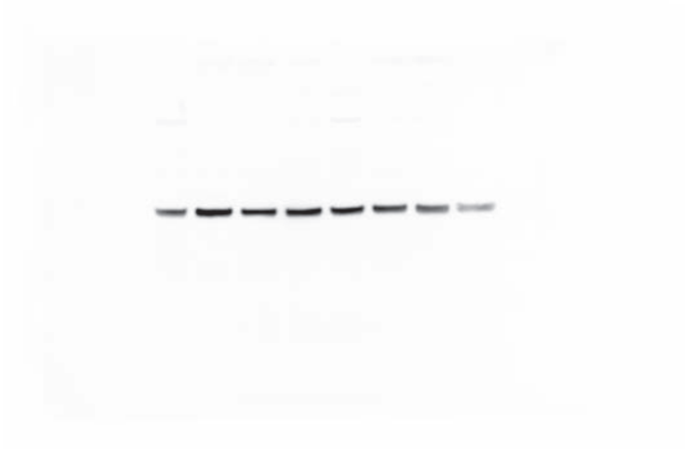

Supplementary Figure 3b - α - K_V7.1 (left) ; α - KCNE1 (right)

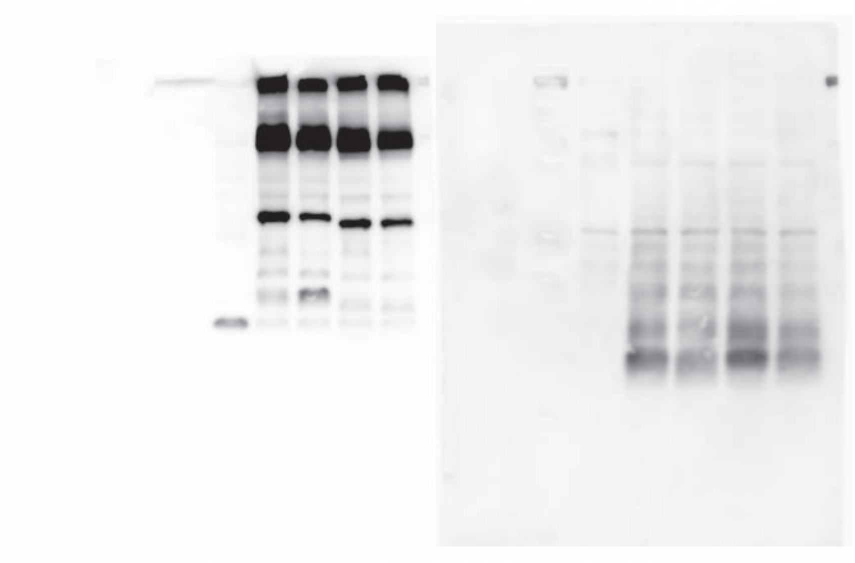

Supplementary Figure 3c - α - K_V7.1

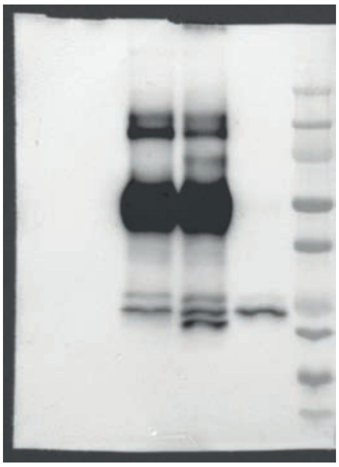

Supplement: Supplementary file 1 — Supplementary Information [file 42003_2018_162_MOESM1_ESM.pdf]
